# Impact of ABCDE bundle nursing on pediatric respiratory distress syndrome under nasal continuous positive airway pressure therapy

**DOI:** 10.12669/pjms.41.7.12275

**Published:** 2025-07

**Authors:** Wenya Wei, Weiyue Wang, Yanna Du, Jianhua Liu, Yuhui Cui

**Affiliations:** 1 Wenya Wei Department of Respiratory, Children’s Hospital of Hebei Province, Hebei Provincial Clinical Research Center for Child Health and Disease, Hebei Provincial Medical Key Discipline, Shijiazhuang, Hebei Province 050000, P.R. China; 2 Weiyue Wang Department of Cardiology, Heibei General Hospital, Shijiazhuang, Hebei Province 050000, P.R. China; 3 Yanna Du Department of Outpatient, Children’s Hospital of Hebei Province, Hebei Provincial Clinical Research Center for Child Health and Disease, Hebei Provincial Medical Key Discipline, Shijiazhuang, Hebei Province 050000, P.R. China; 4 Jianhua Liu Department of Respiratory, Children’s Hospital of Hebei Province, Hebei Provincial Clinical Research Center for Child Health and Disease, Hebei Provincial Medical Key Discipline, Shijiazhuang, Hebei Province 050000, P.R. China; 5 Yuhui Cui Department of Respiratory, Children’s Hospital of Hebei Province, Hebei Provincial Clinical Research Center for Child Health and Disease, Hebei Provincial Medical Key Discipline, Shijiazhuang, Hebei Province 050000, P.R. China

**Keywords:** ABCDE bundle, Children, Nursing, Nasal continuous positive airway pressure, Respiratory distress syndrome

## Abstract

**Objective:**

This study aimed to evaluate the effect of ABCDE nursing on children with respiratory distress syndrome (RDS) receiving nasal continuous positive airway pressure (nCPAP).

**Methods:**

This retrospective cohort study was conducted at Hebei Children’s Hospital and included records of 200 children with RDS who received nCPAP from January 2021 to December 2022. Among them, 100 patients who received ABCDE bundled care (ABCDE group) were matched with the cohort receiving standardized care (control group) in a 1:1 ratio. The primary outcome of interest was hospitalization time, and the secondary outcomes included oxygen therapy duration, blood gas status, incidence of complications, and satisfaction with care provided by the patient’s family.

**Results:**

The oxygen therapy time and hospitalization duration in the ABCDE group were shorter than those in the control group (P<0.05). After intervention, the blood gas status of both groups improved and was considerably better in the ABCDE group than the control group (P<0.05). The total incidence of complications in the ABCDE group was lower (P<0.05), and the satisfaction of family members with the nursing care was higher than in the control group (P<0.05).

**Conclusions:**

ABCDE-based nursing intervention for RDS children receiving nCPAP can improve blood gas status, shorten hospitalization time, reduce the risk of complications, and increase family satisfaction with the nursing care.

## INTRODUCTION

Nasal continuous positive airway pressure (nCPAP) has become an essential method of treating children with pediatric respiratory distress syndrome (RDS).^1^,^2^ Studies show that nCPAP can effectively improve the respiratory function of pediatric patients, increase oxygenation levels, reduce the psychological and physiological trauma of children, and improve the prognosis, and is associated with markedly lower cost than invasive ventilation.^1^-^3^ However, in general, the recovery of patients who receive nCPAP is impacted by various factors, such as long hospital stays and a higher risk of complications.^1^-^4^ With the continuous development of medical nursing concepts, effective nursing models have become exceedingly popular due to their positive effect on the treatment outcomes of pediatric patients.^5^ Therefore, implementing effective nursing care for children receiving nCPAP is crucial for improving the prognosis.^3^-^5^

Conventional nursing often relies on executing relevant operations based on disease characteristics and departmental requirements, with no systematic and targeted approach, resulting in unsatisfactory outcomes.^5^,^6^ ABCDE bundling is a systematic, collaborative, evidence-based nursing model that includes awakening, breathing trials, delirium management, and early exercise/mobility. This model emphasizes the coherence, integrity, and personalization of the nursing process, providing patients with systematic and continuous high-quality nursing services.^7^,^8^

However, there is currently limited research on the effectiveness of ABCDE bundling in children receiving nCPAP. The aim of this study is to clarify the effectiveness of implementing ABCDE bundle nursing in RDS patients receiving nCPAP and provide a new basis for the nursing mode of the pediatric respiratory ward.

## METHODS

This was a single-center retrospective cohort study conducted at Hebei Children’s Hospital, a tertiary pediatric institution. A total of 200 children diagnosed with respiratory distress syndrome (RDS) and treated with nasal continuous positive airway pressure (nCPAP) between January 2021 and December 2022 were included. Among them, 100 patients who received ABCDE bundled nursing care were matched in a 1:1 ratio with 100 patients who received standardized nursing care. Disease severity was evaluated based on respiratory symptoms and chest radiographs in accordance with pediatric respiratory diagnostic guidelines.

### Ethical Approval:

The study was approved by the Ethics Committee of Hebei Children’s Hospital (Approval No. 2021-EC-148, January 2021).

### Inclusion criteria:

- Meet the diagnostic criteria for RDS, with mild to moderate disease severity.^9^
- Age range from one month to 14 years old.
- Underwent nCPAP treatment.
- Has good autonomous breathing ability.
- Complete clinical data.

### Exclusion criteria:

- Patients with congenital pulmonary hypoplasia or respiratory malformations.
- Patients with coagulation dysfunction.
- Patients with visual and auditory impairments.
- Patients with genetic metabolic disorders.
- Patients with inflammatory infectious diseases.

### Standard nursing:

Comprehensive assessment of the child’s condition, detailed recording of respiratory conditions such as respiratory rate and difficulty in breathing were done. Vital signs such as body temperature, heart rate, and blood pressure were monitored, and the nasal cavity was carefully examined for mucosal congestion, swelling, or excessive secretion. Beds were allocated based on the usage of hospital departments and wards, and standard nursing interventions for the affected children were provided, with specific measures.

### ABCDE bundling:

A bundle intervention group was established. The bundle intervention group consisted of two doctors with more than five years of pediatric ICU (PICU) experience, one respiratory therapist, and two specialist nurses. Standardized training was provided for all group members. The division of labor among members was as follows: *a*. Specialist nurses were responsible for daily awakening and jointly evaluating the awakening effect or consciousness status with one doctor; *b*. The spontaneous breathing test was jointly conducted by one doctor, one respiratory therapist, and one specialist nurse; *c*. Sedatives were chosen according to medical advice; *d*. Two specialized nurses used the Riker Sedation Agitation Scale (SAS) and BisPectoral Index (BIS) to monitor the level of sedation in patients.

Children with similar degrees of illness were assigned to the same location for intervention. Specific intervention measures are shown in Table-S2.

The primary outcome of interest was the length of hospital stay. The secondary outcomes included:

- Oxygen therapy duration.
- The blood gas status before intervention and during withdrawal, including partial pressure of carbon dioxide (PaCO2), partial pressure of arterial oxygen (PaO2), blood oxygen saturation (SpO2), and PaO2/inspiratory fraction of oxygen (PaO2/FiO2). PaCO2, PaO2, and SpO2 levels were measured using a Cobas b 123 blood gas analyzer (Roche, Switzerland); The FiO2 level was measured by Trilogy 202 (Philips, Netherlands).
- Complications during hospitalization, including bloating, atelectasis, respiratory muscle associated pneumonia, and nasal injury.
- The satisfaction of the patient’s family members with nursing care was evaluated on the day of discharge using the Newcastle Nursing Satisfaction Scale (NSNS), which is a validated instrument with high reliability (Cronbach’s alpha = 0.92), and is widely used in clinical nursing studies. Satisfaction was categorized as follows: <67 = dissatisfaction, 67–85 = moderate satisfaction, >85 = high satisfaction. Moderately and highly satisfied scores were included in the total satisfaction score.^10^

### Statistical analysis:

All data analyses were conducted using SPSS 27.0 software (IBM Corp, Armonk, NY, USA). The Shapiro-Wilk test was used to evaluate the normality of the evaluation data. Normal distribution data were represented by mean ± standard deviation, independent sample t-test was used for inter-group comparison, and paired t-test was used for intra-group comparison before and after intervention. Non normally distributed data were represented by median and interquartile range. Mann-Whitney U test was used for inter-group comparisons, and Wilcoxon signed rank test was used for intra-group comparisons. The count data were represented by the number of cases, using the chi-square test. P<0.05 was considered statistically significant. The sample size was not determined via formal power analysis but was based on available eligible cases during the two years study period, consistent with prior pediatric cohort designs. No missing data were observed in the primary and secondary outcome variables; hence, no imputation techniques were applied.

## RESULTS

**Fig.1:**
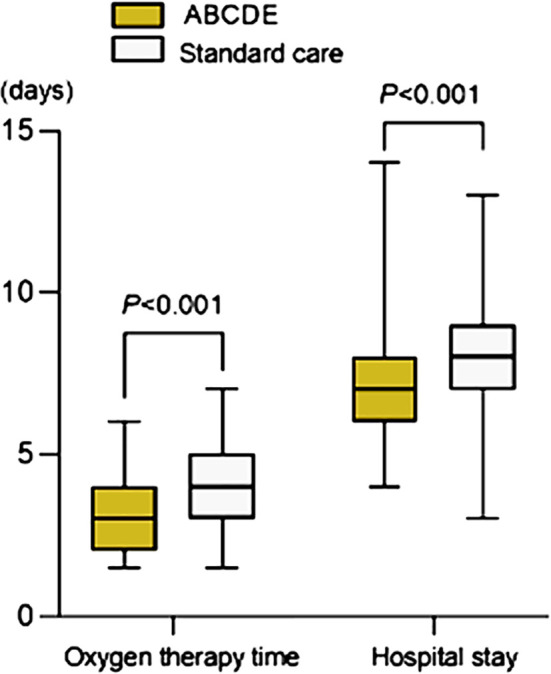
Comparison of oxygen therapy time and hospitalization time between two groups.

This study retrospectively analyzed 200 pediatric patients (107 males and 93 females) aged 0.7-14 years, with a median age of 5.95 (4.5-8). There was no statistically significant difference in baseline data between the two groups (P>0.05) (Table-I). Oxygen therapy time and hospitalization time in the ABCDE group were significantly shorter than those in the control group (both P<0.05) Fig.1.

**Table-I:** Comparison of baseline data between two groups.

Variables	ABCDE group (n=100)	Control group(n=100)	t/χ^2^	P
Age (years), M(P25/P75)	6.3 (4.5-8)	5.85 (4.55-7)	-0.974	0.33
Sex, n (%)			0.181	0.671
Male	52 (52.0)	55 (55.0)		
Female	48 (48.0)	45 (45.0)		
BMI (kg/m²), M(P25/P75)	18.65 (16.5-20.5)	18.4 (16.7-20.25)	-0.882	0.378
Degree of illness, n (%)			0.183	0.669
Mild	42 (42.0)	45 (45.0)		
Moderate	58 (58.0)	55 (55.0)		

Before intervention, the two groups had no significant difference in the levels of PaCO2, PaO2, SpO2, and PaO2/FiO2 (all P>0.05). After intervention, both groups showed a decrease in PaCO2, while PaO2, SpO2, and PaO2/FiO2 increased compared to pre-intervention levels. Post-treatment PaCO2 of the ABCDE group was significantly lower than that of the control group, while PaO2, SpO2, and PaO2/FiO2 were higher (P<0.05) (Table-II).

**Table-II:** Comparison of Blood Gas Status between Two Groups.

Variables		ABCDE group(n=100)	Control group (n=100)	Z/t	P
*Before intervention*	PaCO_2_ (mmHg), M(P25/P75)	51(48.5-54.45)	50.65(47.4-54.3)	-1.169	0.240
	SpO_2_ (%), mean±SD	53.6±8.6	52.5±7.2	1.012	0.313
	PaCO_2_ (mmHg)	82.9±5.7	84.0±4.6	-1.559	0.121
	PaO_2_/FiO_2_, M(P25/P75)	179.5(154.5-208)	183(156.5-221)	-0.567	0.571
*After intervention*	PaCO_2_ (mmHg)	39.3±4.0*	42.8±5.4*	-5.187	<0.001
	PaO_2_ (mmHg)	86.1±7.2*	82.3±6.2*	3.990	<0.001
	SpO_2_ (%), M(P25/P75)	95.3 (90-97.45)*	90.85 (88.25-93.2)*	-4.897	<0.001
	PaO_2_/FiO_2_, M(P25/P75)	305.5(277.5-338)*	246(219.5-284)*	-7.229	<0.001

* Compared to before intervention in the same group; PaCO2, partial pressure of carbon dioxide; PaO2, partial pressure of arterial oxygen; SpO2, blood oxygen saturation; PaO2/FiO2, PaO2/inspiratory fraction of oxygen.

ABCDE bundling was associated with a considerably lower overall incidence of complications compared to the standard nursing approach (6.0% vs 15%, P<0.05). The reported adverse effects in the ABCDE group included two cases of abdominal distension, one case of atelectasis, one case of ventilator-associated pneumonia, and two cases of nasal injury. The control group reported four cases of abdominal distension, four cases of atelectasis, seven cases of ventilator-associated pneumonia, and two cases of nasal injury. Two patients in the control group developed two types of complications. Table-III The rate of family members’ satisfaction with the nursing care in the ABCDE group (93.0%) was significantly higher than in the control group (82.0%) (P<0.05) (Table-IV).

**Table-III:** Comparison of incidence of complications between two groups.

Complications	ABCDE group (n=100)	Control group (n=100)	χ^2^	P
Abdominal distention, n(%)	2 (2.0)	4 (4.0)	0.687	0.678^b^
Atelectasis, n(%)	1 (1.0)	4 (4.0)	1.846	0.365^b^
Ventilator-associated pneumonia, n(%)	1 (1.0)	7 (7.0)	4.688	0.071^b^
Nose injury, n(%)	2 (2.0)	2 (2.0)	0 (0)	1.000^b^
Total incidence rate, n(%)	6 (6.0)	15 (15.0)	4.31	0.038

b: Continuity-corrected Chi-square test was used for expected frequencies less than 5.

**Table-IV:** Comparison of Satisfaction with Nursing Care of family members of two groups of pediatric patients.

Group	n	Very Satisfied	Moderately satisfied	Dissatisfied	Total satisfaction
ABCDE group	100	56 (56.0)	37 (37.0)	7 (7.0)	93 (93.0)
Control group	100	40 (40.0)	42 (42.0)	18 (18.0)	84 (82.0)
*χ^2^*					5.531
*P*					0.019

## DISCUSSION

This study compared the application efficacy of conventional nursing and ABCDE bundle nursing intervention in children with RDS treated with nCPAP. The results showed that ABCDE bundle nursing intervention was associated with better outcomes in terms of oxygen therapy time, length of hospital stay, improvement in blood gas status, overall incidence of complications, and family satisfaction with nursing care. These results indicate that ABCDE bundled care significantly benefits RDS patients receiving nCPAP treatment.

Although ABCDE bundled care was initially designed for ICU patients, its core principles include awakening, breathing trials, delirium management, early exercise/mobility, etc.^7^,^8^,^11^ Notably, this study included children with mild to moderate RDS who necessitate fewer awakening procedures and only require daily assessments of consciousness status and respiratory function.

Mechanical ventilation poses certain risks to patients,^12^ especially for children, who are more prone to developing ventilator-associated pneumonia and cardiovascular complications, which can prolong hospitalization time.^13^ ABCDE bundling covers multiple stages, from assessment to intervention adjustment, reflecting a holistic nursing approach.^11^ In contrast with the traditional nursing approach, ABCDE bundling organically addresses multiple aspects of the child’s condition and care needs, such as breathing, circulation, medication, nutrition, etc., thus providing patients with comprehensive nursing support and shortening hospitalization times.^11^ Chen et al.^14^ found that ABCDE bundle therapy improved patient prognosis, significantly shortening ICU stay, which is consistent with the results of this study.

This study also indicate that ABCDE bundle intervention is associated with better levels of PaCO_2_, PaO_2_, SpO_2_, PaO _2_/FiO_2_ compared to the standard care approach. These improvements are clinically meaningful, as better oxygenation (increased PaO_2_, and SpO_2_) and reduced CO_2_ retention (decreased PaCO_2_) are known to correlate with faster clinical recovery and lower respiratory workload in pediatric RDS. We may speculate that the multidisciplinary approach of the ABCDE bundling results in optimized ventilation strategies and improves patients’ blood gas status, which is consistent with the previous research.^15^ Additionally, numerous studies indicate that autonomous respiration testing is key to optimizing ventilation and oxygenation.^16^,^17^ It is plausible that by monitoring respiratory rate, heart rate, and blood gas levels, the ABCDE bundling approach optimizes CPAP parameter settings to promote early weaning and shorten oxygen therapy time. Together with the previous research showing that early activity promotes lung function recovery and improves oxygenation status,^18^ this study confirms that the ABCDE bundling approach is beneficial for normalizing oxygenation status of RDS patients receiving nCPAP treatment.

The total incidence of complications in the ABCDE group (6.0%) was significantly lower than that in the standard care group (15.0%). This indicates the effectiveness of centralized nursing management in preventing complications. In agreement with these results, Casey et al.^19^ found that in routine nursing, children are more likely to develop complications due to the lack of systematic and comprehensive nursing interventions. The ABCDE model can identify and address potential risk factors in a timely manner through comprehensive evaluation and multi-link nursing interventions.^8^,^19^ Identifying high-risk factors for individual complications early in the evaluation process and reviewing medication and nutrition plans may prevent infections and injuries caused by improper airway care.^19^,^20^ Other studies have shown that in the evaluation process of the ABCDE model, personalized nursing plans can be developed based on the patient’s specific situation.^21^,^22^ Due to the differences in the condition of patients and their family environment, personalized care can better meet individual special needs and ensure the effectiveness of interventions.^22^

The satisfaction of sick children’s families is an important indicator of nursing care quality.^23^ This study showed that the ABCDE bundling care leads to faster improvement of the patient’s condition and shorter length of hospital stay, which directly reduces the concerns of the family members and improves their satisfaction with the overall care, in agreement with previous research.^22^-^24^ This study contributes novel evidence for the implementation of ABCDE bundle in general pediatric respiratory settings, with strengths including matched design and comprehensive outcome assessment.

### Limitations:

This retrospective study was conducted in a tertiary center as a retrospective cohort study with a small sample size. The severity of the children’s conditions was mild or moderate, and they received treatment in a regular ward. There were no follow-up statistics on adverse events after discharge. Additionally, the study only included children with RDS who received non-invasive continuous positive airway pressure ventilation. However, respiratory dysfunction caused by different diseases may have different responses to nursing models. Future research is needed to study the efficacy of ABCDE bundled care in children with different types of diseases, such as congenital heart disease combined with respiratory dysfunction or respiratory dysfunction caused by pneumonia.

## CONCLUSION

Compared with standardized nursing, ABCDE bundling nursing can improve blood gas status, shorten oxygen therapy and hospitalization time in RDS children receiving nCPAP. ABCDE approach is associated with the reduced risk of complications, and higher family satisfaction. Although initially designed for ICU patients, ABCDE bundled care may also be applicable to patients in general wards after appropriate adjustments. These results support the integration of ABCDE-based nursing practices into pediatric respiratory wards to promote early recovery and reduce complications. High-quality, large-scale prospective studies are needed in the future to validate the results of this study. Future research should explore ABCDE nursing in diverse pediatric respiratory illnesses and assess its feasibility in low-resource settings and community hospitals.

## Authors’ contributions:

**Wenya Wei and Weiyue Wang:** Literature Search, study design and manuscript writing.

**YD, JL and YC:** Data collection, data analysis and interpretation. Critical review.

**Wenya Wei and Weiyue Wang:** Manuscript revision and validation and is responsible for the integrity of the study.

All authors have read and approved the final manuscript.
